# Mono-Doped and Co-Doped Nanostructured Hematite for Improved Photoelectrochemical Water Splitting

**DOI:** 10.3390/nano12030366

**Published:** 2022-01-24

**Authors:** Justine Sageka Nyarige, Alexander T. Paradzah, Tjaart P. J. Krüger, Mmantsae Diale

**Affiliations:** Department of Physics, University of Pretoria, Private Bag X20, Hatfield, Pretoria 0028, South Africa; justine.nyarige@up.ac.za (J.S.N.); paradzah.alex@gmail.com (A.T.P.); tjaart.kruger@up.ac.za (T.P.J.K.)

**Keywords:** hematite nanoparticles, doping, chemical spray pyrolysis, photocurrent, water-splitting, transient absorption spectroscopy

## Abstract

In this study, zinc-doped (α-Fe${}_{2}$O${}_{3}$:Zn), silver-doped (α-Fe${}_{2}$O${}_{3}$:Ag) and zinc/silver co-doped hematite (α-Fe${}_{2}$O${}_{3}$:Zn/Ag) nanostructures were synthesized by spray pyrolysis. The synthesized nanostructures were used as photoanodes in the photoelectrochemical (PEC) cell for water-splitting. A significant improvement in photocurrent density of 0.470 mAcm${}^{-2}$ at 1.23 V vs. reversible hydrogen electrode (RHE) was recorded for α-Fe${}_{2}$O${}_{3}$:Zn/Ag. The α-Fe${}_{2}$O${}_{3}$:Ag, α-Fe${}_{2}$O${}_{3}$:Zn and pristine hematite samples produced photocurrent densities of 0.270, 0.160, and 0.033 mAcm${}^{-2}$, respectively. Mott–Schottky analysis showed that α-Fe${}_{2}$O${}_{3}$:Zn/Ag had the highest free carrier density of 8.75 × 10${}^{20}$ cm${}^{-3}$, while pristine α-Fe${}_{2}$O${}_{3}$, α-Fe${}_{2}$O${}_{3}$:Zn, α-Fe${}_{2}$O${}_{3}$:Ag had carrier densities of 1.57 × 10${}^{19}$, 5.63 × 10${}^{20}$, and 6.91 × 10${}^{20}$ cm${}^{-3}$, respectively. Electrochemical impedance spectra revealed a low impedance for α-Fe${}_{2}$O${}_{3}$:Zn/Ag. X-ray diffraction confirmed the rhombohedral corundum structure of hematite. Scanning electron microscopy micrographs, on the other hand, showed uniformly distributed grains with an average size of <30 nm. The films were absorbing in the visible region with an absorption onset ranging from 652 to 590 nm, corresponding to a bandgap range of 1.9 to 2.1 eV. Global analysis of ultrafast transient absorption spectroscopy data revealed four decay lifetimes, with a reduction in the electron-hole recombination rate of the doped samples on a timescale of tens of picoseconds.

## 1. Introduction

**Figure 1:**
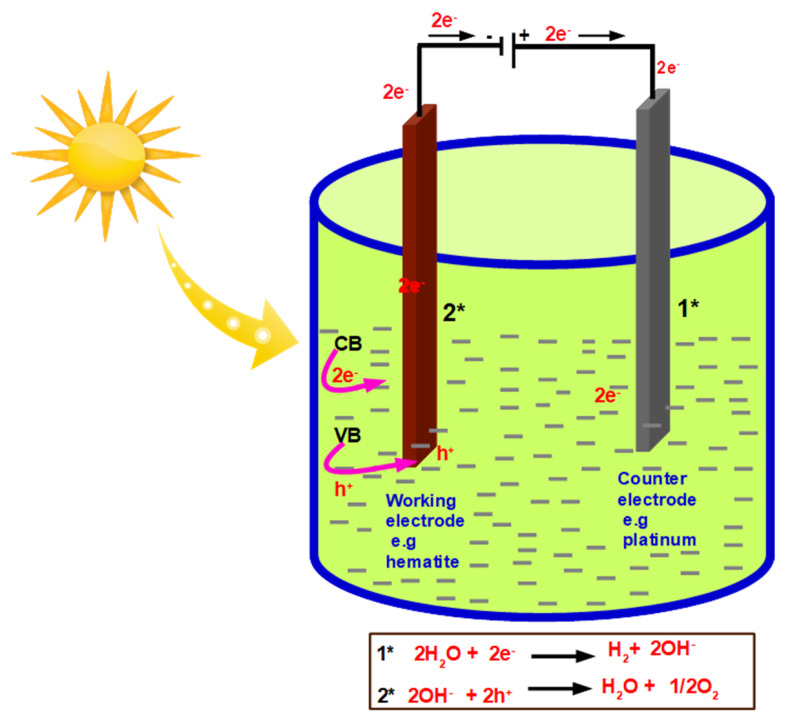
Schematic of the PEC cell for water splitting setup.

Solar energy is one of the alternative solutions to the existing gap of energy demand if proper utilization is done. Global energy consumption is projected to rise by 28% from 575 quadrillion British thermal units (Btu) in 2015 to 736 quadrillion Btu in 2040 [1]. Harvesting of 0.04% of the solar energy to produce 50 TW will be sufficient to meet the world energy demand. One of the effective and clean approaches for the production of clean energy is solar-driven water-splitting, where hydrogen (H${}_{2}$) and oxygen (O${}_{2}$) gases are released [2]. During this process, sunlight is absorbed using a suitable semiconductor producing electron-hole (e${}^{-}$h${}^{+}$) pairs. The holes and electrons are separated and used for oxidation of H${}_{2}$O and reduction of H${}^{-}$ to produce oxygen and hydrogen gases, respectively. In comparison with other fossil fuels, H${}_{2}$ can produce high efficiency with its fuel being environmentally benign [3]. The principle of a PEC cell for water-splitting is presented in Figure 1.

Different semiconductors have been employed in PEC applications. To ensure efficient water-splitting, a suitable semiconductor should have an appropriate bandgap (<2.5 eV), and good conductivity [4]. However, finding a semiconductor that meets these two conditions has been a long-standing challenge. Hematite (α-Fe${}_{2}$O${}_{3}$) has been used as a photoanode due to its small bandgap (1.9–2.2 eV) that absorbs 40% of the solar spectrum in the visible region [5]. Additionally, hematite is chemically stable and relatively abundant [6]. Despite these advantages, α-Fe${}_{2}$O${}_{3}$ has some shortcomings that include poor conductivity and fast electron-hole recombination. In addition, the conduction band edge of hematite is below the H${}^{+}$/H${}_{2}$ potential, hence an external bias is needed to ensure water reduction.

Due to hematite’s poor conductivity and short e${}^{-}$h${}^{+}$ diffusion lengths, several methods have been explored to possibly improve the photocurrent production. One of these approaches includes nanostructuring, which was first reported by Fitzmaurice et al. with ruthenium-based complexes on TiO${}_{2}$ [7]. The nanostructures provide a large semiconductor–electrolyte interface in which the redox reactions take place to enable the charge separation. Similarly, nanostructured and ultrathin films have been reported before as one of the ways to address the short diffusion length, which is one of the limitations of hematite in PEC water-splitting. The bandgap of the photoelectrode materials can be manipulated due to the quantum confinement effect resulting from nanostructuring [8]. Underlayers and overlayers have also been used to reduce surface recombination and enhance charge separation [9,10,11]. Doping of hematite with metals can increase the charge carrier density and accelerate the surface oxidation reaction, hence improving the photocurrent [12]. Metal ion doping, on the other hand, can also create recombination sites, thereby creating another energy level that might hinder the electron transfer [13]. Through doping, PEC water oxidation/reduction efficiency is enhanced due to the reduction in the crystalline disorder [14].

To study the electron-hole lifetimes and exciton dynamics of hematite nanostructured thin films, several transient absorption spectroscopy (TAS) studies have been conducted [11,15,16,17]. TAS on pristine and doped hematite can help in determining the effect of doping on the electron-hole recombination [16,18]. The charge carrier dynamics on passivated hematite surfaces have been reported by Barroso et al. on microsecond and second timescales [9]. The lifetime of photogenerated holes was increased as a result of cobalt phosphate. From their study, doping and heterojunctioning can therefore enhance the lifetime of the charge carries and this is one of the motivations of this study. The effect of the applied bias on charge recombination using TAS has also been reported [19]. In addition, a study by Liam et al. reported little change in the charge recombination in nanostructures with large grain sizes. Despite other reports on the role of dopants on the charge carrier dynamics and recombination, limited research has been conducted on the effect of surface doping on the charge carrier dynamics of α-Fe${}_{2}$O${}_{3}$:Zn, α-Fe${}_{2}$O${}_{3}$:Ag, and α-Fe${}_{2}$O${}_{3}$:Zn/Ag nanoparticles prepared using spray pyrolysis. This research provides a deeper understanding of electron-hole recombination on doped hematite.

In this study, spray pyrolysis was used to synthesize α-Fe${}_{2}$O${}_{3}$:Zn, α-Fe${}_{2}$O${}_{3}$:Ag and α-Fe${}_{2}$O${}_{3}$:Zn/Ag nanoparticles at a deposition temperature of 430 ${}^{{}^{\circ}}$C. From the decay lifetimes, femtosecond and picosecond timescales obtained by ultrafast transient absorption spectroscopy measurements of doped hematite reported reduction in the electron-hole recombination. Current-density and voltage measurements of α-Fe${}_{2}$O${}_{3}$:Zn/Ag reported an improvement in photocurrent density from 0.040 mAcm${}^{-2}$ for pristine α-Fe${}_{2}$O${}_{3}$ to 0.470 mAcm${}^{-2}$ for α-Fe${}_{2}$O${}_{3}$:Zn/Ag at 1.23 V vs. RHE.

## 2. Experimental Details

**Figure 2:**
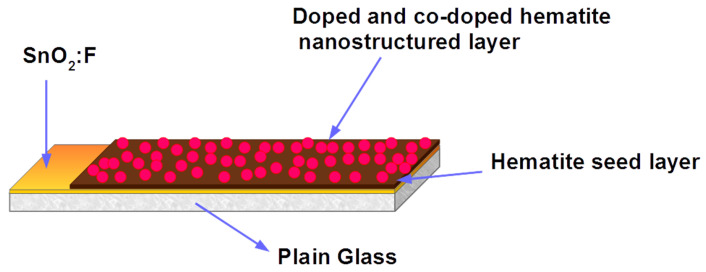
Architecture of nanostructured α-Fe${}_{2}$O${}_{3}$:Zn, α-Fe${}_{2}$O${}_{3}$:Ag and α-Fe${}_{2}$O${}_{3}$:Zn/Ag samples prepared by chemical spray pyrolysis at 430 ${}^{{}^{\circ}}$C deposition temperature.

Chemical spray pyrolysis (CSP) was used in the synthesis of hematite nanostructures, as described in our previous work [20,21]. The samples were prepared on a fluorine-doped tin oxide (SnO${}_{2}$:F) glass substrate using 50 mM of the dissolved iron nitrate nonahydrate, Fe(NO${}_{3})$${}_{3}$.9H${}_{2}$O (Sigma Aldrich, Johannesburg, South Africa) as the precursor and deionized water (DI) as the solvent [22]. A nozzle-to-substrate height of 20 cm, spray pressure of 2.2 × 10${}^{-5}$ Pa, and 0.2 mm nozzle diameter were all kept constant during thin film deposition. Nitrogen gas was used as the carrier gas. The pristine samples were calcinated at 500 ${}^{{}^{\circ}}$C for 1 h and left to cool naturally to room temperature. These samples were used as seed layers for the surface-doping of Ag, Zn, and Zn/Ag. α-Fe${}_{2}$O${}_{3}$:Zn, α-Fe${}_{2}$O${}_{3}$:Ag, and α-Fe${}_{2}$O${}_{3}$:Zn/Ag samples were prepared using zinc nitrate hexahydrate (Zn(NO${}_{3}$)${}_{2}$.6H${}_{2}$O, Sigma Aldrich, South Africa) and silver nitrate (Ag(NO)${}_{3}$, Sigma Aldrich, Johannesburg, South Africa) as the precursor dopants, with a doping concentration of 0.5 wt%. The dopant precursor was then mixed with 50 mM of Fe(NO${}_{3})$.9H${}_{2}$O for 10 min at room temperature to obtain a homogeneous solution. For the surface co-doped samples, 0.5 wt % of (Ag(NO)${}_{3}$), Zn(NO${}_{3}$)${}_{2}$.6H${}_{2}$O prepared separately were added to a beaker containing 50 mM solution of Fe(NO${}_{3})$.9H${}_{2}$O, and stirred to obtain a uniform solution. The solution was then sprayed onto the annealed hematite samples using CSP for 20 s at 430 ${}^{{}^{\circ}}$C to create a thin, nanostructured, doped hematite layer on top of the seed layer. The surface doped and co-doped samples were finally annealed at 500 ${}^{{}^{\circ}}$C and left to cool for 12 h at room temperature. The samples were annealed separately to avoid possible contamination at high temperatures. Figure 2 presents the architecture of as-prepared surface doped and pristine hematite samples.

A field-emission scanning electron microscope (Zeiss Crossbeam 540 FESEM-Microscopes) operating at 2 kV was used to study the surface morphology of the nanostructures. The crystal structures of the pristine, doped, and co-doped samples were confirmed using a Bruker D2 Phaser X-ray diffractometer (XRD) with CuK${}_{\alpha }$ radiation, a 0.15418 nm source, scanning speed of $0.05^{{}^{\circ}}$ per minute and a 2θ range of $20^{{}^{\circ}}$–$70^{{}^{\circ}}$. A Cary 100 Bio UV-Vis spectrophotometer was used to measure the optical properties of these doped and pristine hematite thin films. Electrochemical measurements were carried out using a potentiostat (VersaSTAT 3F potentiostat from Princeton Applied Research). The prepared pristine hematite, α-Fe${}_{2}$O${}_{3}$:Zn, α-Fe${}_{2}$O${}_{3}$:Ag and α-Fe${}_{2}$O${}_{3}$:Zn/Ag were used as working electrodes. For the counter-electrode, a platinum mesh was used, while 3.0 M KCl saturated Ag/AgCl was our reference electrode. Linear Scan Voltammetry (LSV) measurements were performed using 1.0 M NaOH, pH 13.8 as the electrolyte, with a scan rate of 0.05 mVs${}^{-1}$ in the voltage range of 0 to 1 V vs. Ag/AgCl. Dark measurements were first done for 5 min before running the experiment. A 0.49 cm${}^{2}$ area of the pristine and doped hematite samples was irradiated using a solar simulator (Newport LSC 100 W Xenon lamp), with AM 1.5 G (100 mW cm${}^{-2}$) spectrally corrected by filters. The potentials obtained were thereafter converted to a reversible hydrogen electrode (RHE) using the Nernst Equation (1) [20]:

(1) $$E_{RHE}=E_{\left(Ag/AgCl\right)}+0.059PH+E_{\left(Ag/AgCl\right)}^{{}^{\circ}},$$

where *E*${}^{{}^{\circ}}$${}_{\left(Ag/AgCl\right)}$ is 0.205 V at 25${}^{{}^{\circ}}$, and *E*${}_{\left(Ag/AgCl\right)}$ is the potential against the reference electrode (Ag/AgCl) measured experimentally. A potential that ranged from −0.2 V to 0.5 V vs. Ag/AgCl at a frequency of 10 kHz was used to perform Mott–Schottky and impedance measurements under dark conditions.

**Figure 3:**
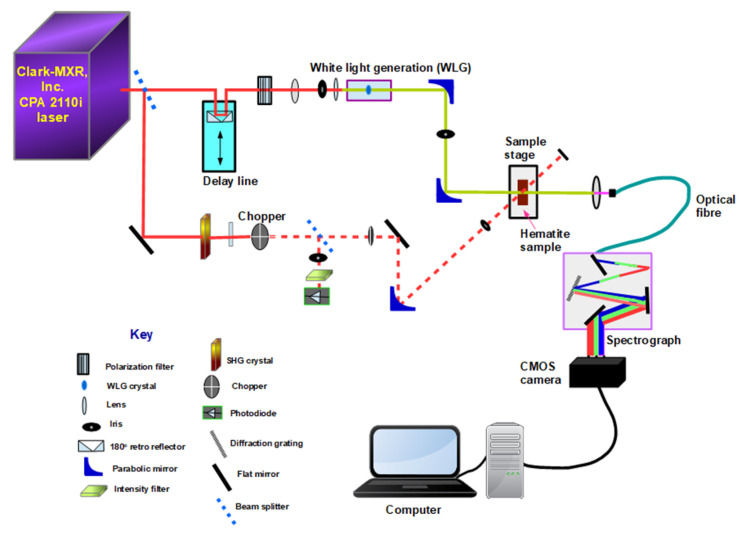
Schematic diagram of the femtosecond transient absorption spectroscopy setup used for the pump-probe measurements of pristine α-Fe${}_{2}$O${}_{3}$, α-Fe${}_{2}$O${}_{3}$:Zn, α-Fe${}_{2}$O${}_{3}$:Ag and α-Fe${}_{2}$O${}_{3}$:Zn/Ag samples.

Ultrafast transient absorbance spectroscopy measurements were performed on all the prepared samples to study the lifetimes of the charge carriers and the exciton dynamics using a Ti:Sapphire chirped-pulse amplified laser source (Clark-MXR) operating at a 1 kHz repetition rate, with a peak output wavelength of 775 nm, a pulse width of 150 fs, and average peak power of 800 mW. The output beam was split into two (pump and probe) using a beam splitter. Approximately 565 mW was used as the probe beam, while 235 mW was used as the pump beam. The pump beam was sent through a beta barium borate (BBO) crystal for frequency doubling to produce a 387.5 nm centered beam having a FWHM bandwidth of 4 nm. A chopper placed in the pump beam was used to enable “pumped” and “unpumped” measurements. The probe beam was passed through a 2 ns optical delay line. Thereafter, the probe beam was attenuated and focused on a sapphire crystal to produce a white light continuum in the 430–700 nm wavelength range. The probe and pump beams were made to overlap spatially and temporally inside the sample. At the sample, the power of the pump beam was 25 mW. Beyond the sample, the pump beam was blocked, while the probe beam was focused into an optical fiber and dispersed onto a CMOS sensor (Synertronic Designs) to capture the transmitted spectra. Finally, Glotaran was used to perform global analysis of the data using a four-component sequential model [23]. The number of model compartments was determined by singular value decomposition (SVD) of the raw data. From the analysis model, evolution-associated difference spectra (EADS) and corresponding decay lifetimes were obtained. The femtosecond pump-probe experimental setup is depicted in Figure 3.

## 3. Results and Discussion

### 3.1. X-ray Diffraction

**Figure 4:**
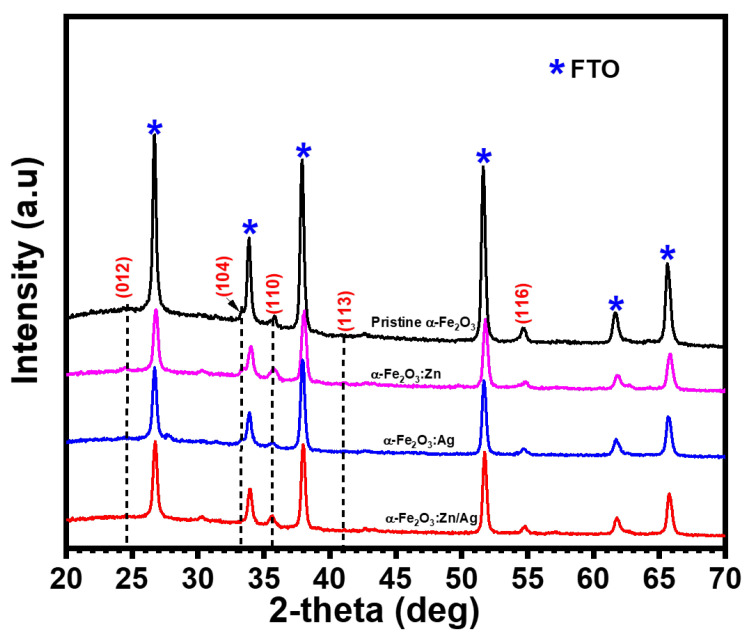
XRD patterns of pristine α-Fe${}_{2}$O${}_{3}$, α-Fe${}_{2}$O${}_{3}$:Zn, α-Fe${}_{2}$O${}_{3}$:Ag and α-Fe${}_{2}$O${}_{3}$:Zn/Ag samples prepared by chemical spray pyrolysis at 430 ${}^{{}^{\circ}}$C.

The crystal structure of α-Fe${}_{2}$O${}_{3}$:Zn, α-Fe${}_{2}$O${}_{3}$:Ag and α-Fe${}_{2}$O${}_{3}$:Zn/Ag were confirmed using XRD (Figure 4). The intensity of the main phases of hematite (104) and (110) indexed at 33.2${}^{{}^{\circ}}$ and 35.5${}^{{}^{\circ}}$, respectively, matched the corundum rhombohedral structure of hematite, with *a* = 5.075 Å and *c* = 13.748 Å (JCPDS file 33-0664) and was observed for all the samples.

The peak intensities of the doped samples did not decrease in relation to undoped hematite. The (110) phase has a relatively higher intensity as compared to (104) peak of rhombohedral structure for both the undoped and doped hematite, an indication that the doped samples still had a vertical orientation of their (001) plane with respect to the substrate. This orientation has been reported to be favorable to photoanode activity [24]. Naghmehalsadat et al. have reported similar results on Zn- and Ti-doped hematite for photoelectrochemical water oxidation using an electrodeposition technique [25]. JCPDS file no. 33-0664 was also used to confirm the indexing of all other 2-theta planes. Besides, other crystal structures of Zn and Ag were not detected in our XRD study. Doping of hematite led to an increase in the lattice parameters. This could be due to the increase in the ionic radius of Ag${}^{+}$(0.115 nm) and Zn${}^{2+}$(0.074 nm) compared to Fe${}^{3+}$(0.064 nm) and O${}^{2-}$(0.140 nm). Additionally, there could be a possible increase in the Coulombic repulsion between the ion charges during doping [26].

### 3.2. Morphology of Hematite Nanoparticles

**Figure 5:**
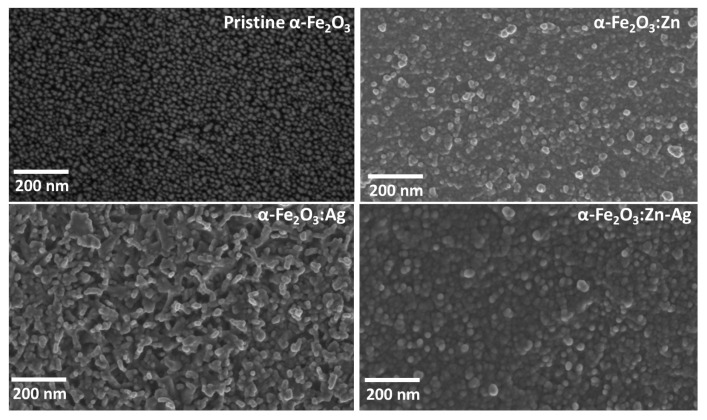
FESEM images of pristine α-Fe${}_{2}$O${}_{3}$, α-Fe${}_{2}$O${}_{3}$:Zn, α-Fe${}_{2}$O${}_{3}$:Ag and α-Fe${}_{2}$O${}_{3}$:Zn/Ag samples.

A FESEM analysis in Figure 5 shows a nanoporous nanoparticle distribution on the surface with a uniform distribution of the grain sizes for all the samples. For α-Fe${}_{2}$O${}_{3}$, the grains were rod-like and the pores were of a similar size. For all the samples the grain sizes were <30 nm as determined using ImageJ software. There was a uniform distribution of the grain sizes over all the samples. However, for the α-Fe${}_{2}$O${}_{3}$:Ag sample, there was an observed rod-like grain with pores that were almost having the same diameter. The differences in the grain sizes could possibly arise from different nucleation media of the dopant precursors used. A small grain size is advantageous considering that hematite suffers from short electron-hole diffusion lengths [16]. This enables more charge carriers to reach the surface before recombination takes place. The film thickness of 545 nm, 497 nm, 462 nm, and 433 nm were estimated from the cross-section using FESEM. Due to the poor absorption of hematite, a thickness ranging from 400 to 500 nm is required for complete absorption to take place [27].

### 3.3. Optical Properties

**Figure 6:**
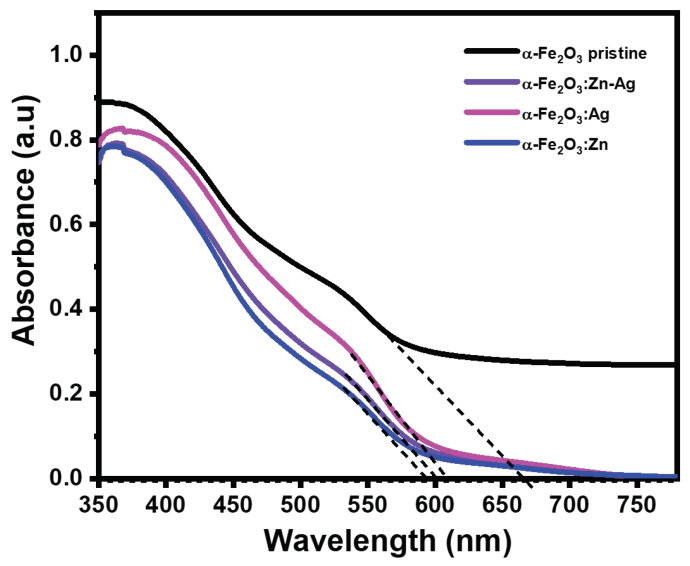
Optical absorption of pristine α-Fe${}_{2}$O${}_{3}$, α-Fe${}_{2}$O${}_{3}$:Zn, α-Fe${}_{2}$O${}_{3}$:Ag, and α-Fe${}_{2}$O${}_{3}$:Zn/Ag samples prepared by spray pyrolysis.

The optical absorbance of the α-Fe${}_{2}$O${}_{3}$:Zn, α-Fe${}_{2}$O${}_{3}$:Ag and α-Fe${}_{2}$O${}_{3}$:Zn/Ag samples was measured using UV-Vis spectroscopy. The samples absorbed in the visible region, with an onset absorbance in the range of 592 nm to 665 nm as shown in Figure 6. The absorbance of the doped samples was slightly blue-shifted in comparison to pristine hematite. This could be due to a decrease in thickness and doping effect. Research has shown that, unlike thick films that present a single transition, thinner films like the ones obtained in this study can have three transitions [3]. These transitions indicate the presence of intermediate energy levels, which signify electrons trapped closer to the conduction band [3]. Stress caused by a strong interaction between the substrate and the film could have led to the origin of the intermediate levels [28]. The bandgaps of these thin films were estimated by extrapolating the linear part of the absorption spectra close to the onset of absorbance and converting to eV using the relation,

(2) $$E_{g(eV)}=\frac{1240}{\lambda (nm)},$$

where λ is the wavelength where the extrapolated linear absorption intersects the *x*-axis. Absorption in the visible range is due to Fe${}^{3+}$ d-d transitions [29]. Although d-d transitions are spin forbidden, they do occur with a low probability due to spin-orbit coupling [29]. Maximum absorbance for the doped samples was observed around 400 nm. This could be due to ligand to metal charge transfer (LMCT) from the O(2p) orbitals to the Fe${}^{3+}$2t${}_{2g}$ and 3e${}_{g}$ orbitals [30]. However, for pristine hematite the absorbance increases further to the blue of 400 nm. The bandgaps of pristine α-Fe${}_{2}$O${}_{3}$, α-Fe${}_{2}$O${}_{3}$:Zn, α-Fe${}_{2}$O${}_{3}$:Ag and α-Fe${}_{2}$O${}_{3}$:Zn/Ag were obtained as 1.9, 2.1, 2.0 and 2.1 eV, respectively, which fall within the standard hematite bandgap values.

### 3.4. Photocurrent Density Measurements

**Figure 7:**
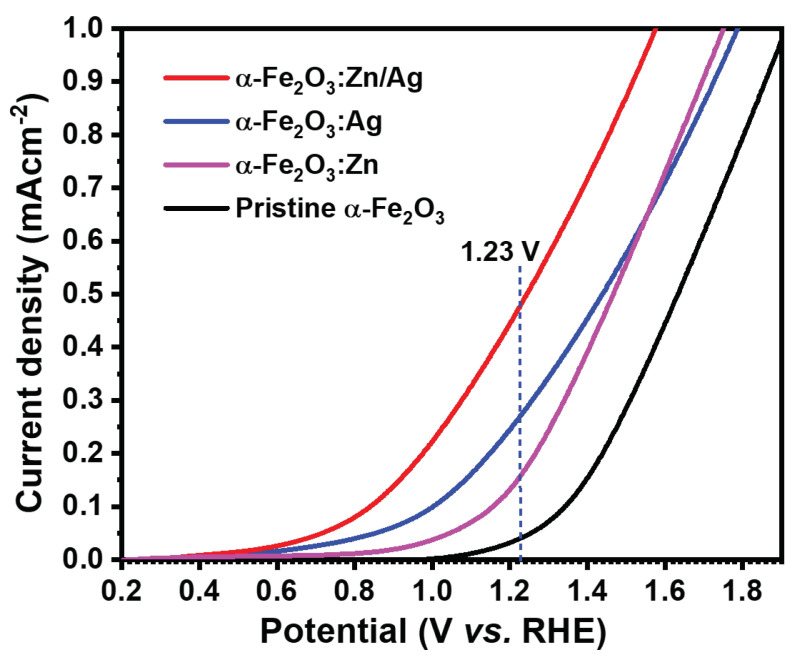
Photocurrent densities of pristine α-Fe${}_{2}$O${}_{3}$, α-Fe${}_{2}$O${}_{3}$:Zn, α-Fe${}_{2}$O${}_{3}$:Ag and α-Fe${}_{2}$O${}_{3}$:Zn/Ag. A scan rate of 50 mVs${}^{-1}$ and 1 M NaOH as electrolyte were used.

**Figure 8:**
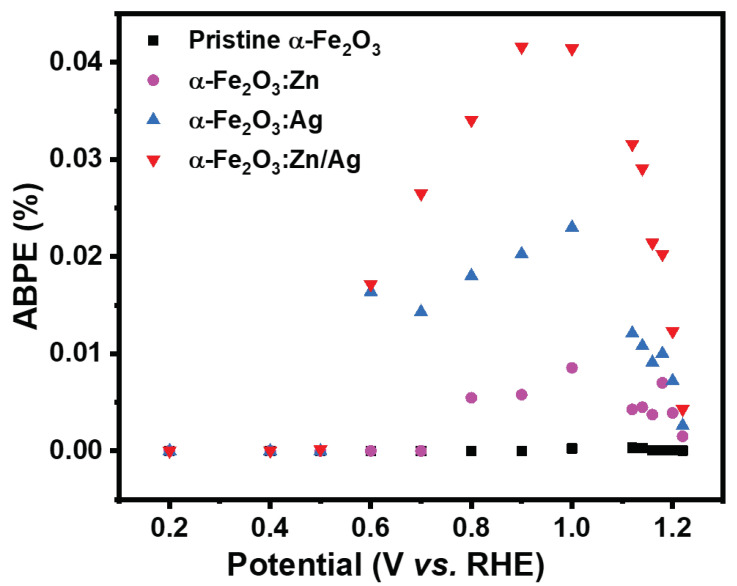
ABPE spectra of pristine α-Fe${}_{2}$O${}_{3}$, α-Fe${}_{2}$O${}_{3}$:Zn, α-Fe${}_{2}$O${}_{3}$:Ag and α-Fe${}_{2}$O${}_{3}$:Zn/Ag for potentials of 0.2 to 1.2 V vs. RHE.

The performance of the electrodes was obtained using a 1 M NaOH electrolyte (pH 13.8), both in the dark and under simulated solar light (AM 1.5 G, 100 mWcm${}^{-2}$). Figure 7 shows current densities of the pristine α-Fe${}_{2}$O${}_{3}$, α-Fe${}_{2}$O${}_{3}$:Zn, α-Fe${}_{2}$O${}_{3}$:Ag, and α-Fe${}_{2}$O${}_{3}$:Zn/Ag samples that were used as photoanodes for PEC water-splitting. Photocurrent densities of 0.033, 0.160, 0.270, and 0.470 mAcm${}^{-2}$ at 1.23 V vs. RHE were obtained for the pristine α-Fe${}_{2}$O${}_{3}$, α-Fe${}_{2}$O${}_{3}$:Zn, α-Fe${}_{2}$O${}_{3}$:Ag and α-Fe${}_{2}$O${}_{3}$:Zn/Ag photoanodes, respectively. There was a significant increase in the photocurrent density of α-Fe${}_{2}$O${}_{3}$:Zn/Ag as compared to all other samples. The J-V curves were used to calculate the applied bias photon to current efficiency (ABPE) below the thermodynamic potential of water oxidation (1.23 V vs. RHE) from a working and reference electrode using the equation:

(3) $$ABPE=\frac{J_{p}\times (1.23-V_{bias})}{P_{in}}\times 100\%,$$

where $J_{p}$ is the photocurrent density (mAcm${}^{-2}$), $V_{bias}$ is the applied voltage between the two electrodes and $P_{in}$ is the incident illumination power density (100 mWcm${}^{-2}$). Figure 8 presents the calculated ABPE efficiency of pristine and doped hematite for a bias potential of 0.2–1.2 V vs. RHE [31,32,33]. It can be seen that the efficiency increased as the potentials were increased for the doped samples. The 1.0 V vs. RHE gave the maximum efficiency of 0.0413, 0.0023, 0.0085, and 0.000253% for α-Fe${}_{2}$O${}_{3}$:Zn/Ag, α-Fe${}_{2}$O${}_{3}$:Ag, α-Fe${}_{2}$O${}_{3}$:Zn, and pristine α-Fe${}_{2}$O${}_{3}$ respectively. The low photocurrent obtained for the pristine hematite could be due to the poor conductivity and is similar to our previous studies [21,22]. The improvement of the current density of the doped samples could be due to the increase in donor density, supporting how the recombination of the photogenerated electrons and holes was significantly reduced as discussed in Section 3.5. On the other hand, the onset potential shifted cathodically by about 750 mV for pristine α-Fe${}_{2}$O${}_{3}$ and α-Fe${}_{2}$O${}_{3}$:Zn/Ag. Another possibility of photocurrent improvement is suppression in the formation of surface traps in α-Fe${}_{2}$O${}_{3}$:Zn, α-Fe${}_{2}$O${}_{3}$:Ag and α-Fe${}_{2}$O${}_{3}$:Zn/Ag, thus enhancing the transient lifetimes [26]. Pristine hematite has low electron mobility, hence high electron-hole recombination, resulting in low photocurrent densities. Higher photocurrents for the doped and codoped hematite were not obtained since we used low doping concentrations. A study by Naghmehalsadat et al. showed that increase in the Zn and Ti doping concentration improved the photocurrents obtained [25]. Additionally, Chen et al., in their study of doping hematite with Zn, revealed a photocathode from photoanode hematite with improved photocurrents [34]. However, in this study, we only used low concentration to study the effect of doping on photoelectrochemical water-splitting. The low doping concentrations used did not change hematite from photoanode to photocathode, as reported in the Mott–Schottky analysis.

### 3.5. Electrochemical Impedance Measurements

**Figure 9:**
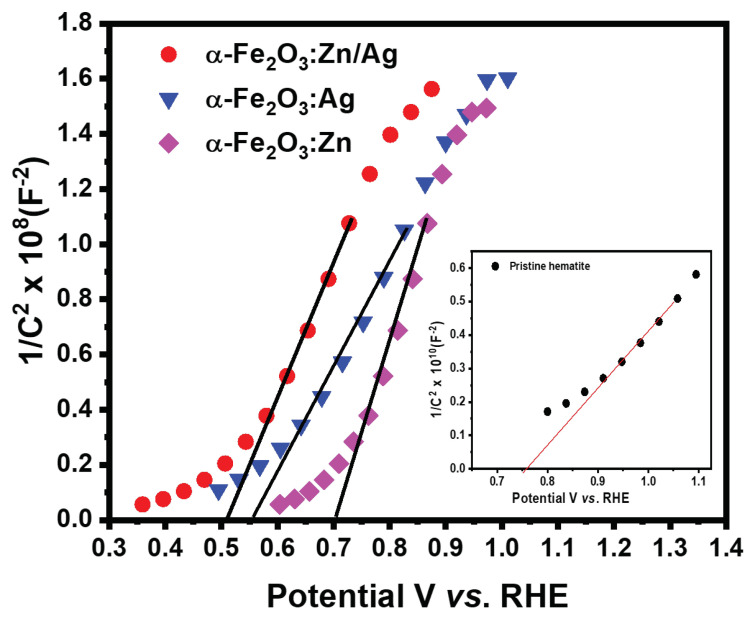
Mott–Schottky curves for pristine α-Fe${}_{2}$O${}_{3}$, α-Fe${}_{2}$O${}_{3}$:Zn, α-Fe${}_{2}$O${}_{3}$:Ag and α-Fe${}_{2}$O${}_{3}$:Zn/Ag in the dark. The inset shows the Mott–Schottky curve of pristine α-Fe${}_{2}$O${}_{3}$. Linear fits (solid lines) of the linear sections of the curves were used to estimate *E*${}_{FB}$ from the x intercept and *N*${}_{D}$ from the slope.

Mott–Schottky (M-S) measurements at 10 kHz were performed on the pristine and doped samples to investigate the electronic properties of the nanostructures. Figure 9 shows that the M-S slopes were all positive, an indication that the doped and co-doped samples are n-type. The carrier density and flat band potentials were estimated using the equation,

(4) $$\frac{1}{C^{2}}=\frac{2}{q\varepsilon \varepsilon_{o}A^{2}N_{D}}\left(E-E_{FB}\right)-\frac{kT}{q},$$

where *C* is the space charge capacitance, *q* is the electron charge, ε is the dielectric constant of hematite [35], ε${}_{o}$ is the dielectric permittivity of vacuum, *A${}^{2}$* is the surface area of the electrode, *N${}_{D}$* is the carrier density, *E* is the applied electrode potential, *E${}_{FB}$* is the flat band potential, *k* is Boltzmann’s constant and *T* is room temperature. The donor density (*N${}_{D}$*) was obtained from the slope of the graph while the onset potential gives the flat band potential (*E${}_{FB}$*).

The flat band potential of the pristine α-Fe${}_{2}$O${}_{3}$, α-Fe${}_{2}$O${}_{3}$:Zn, α-Fe${}_{2}$O${}_{3}$:Ag, and α-Fe${}_{2}$O${}_{3}$:Zn/Ag samples were obtained as 0.76, 0.73, 0.56, and 0.51 V vs. RHE, respectively. The donor densities were obtained as 1.57 × 10${}^{19}$, 5.63 × 10${}^{20}$, 6.91 × 10${}^{20}$, and 8.75 × 10${}^{20}$ cm${}^{-3}$ for the pristine α-Fe${}_{2}$O${}_{3}$, α-Fe${}_{2}$O${}_{3}$:Zn, α-Fe${}_{2}$O${}_{3}$:Ag and α-Fe${}_{2}$O${}_{3}$:Zn/Ag samples, respectively. There was a one order increase in the donor density for α-Fe${}_{2}$O${}_{3}$:Zn/Ag as compared to the pristine α-Fe${}_{2}$O${}_{3}$. This increase in *N*${}_{D}$ and the negative shift of *E${}_{FB}$* could have led to the improvement in the photocurrent. The improved conductivity could have further led to the transfer of surface electrons, reducing the recombination of electrons and holes, hence favoring a low onset potential [36]. The results obtained are consistent with those of Xi et al., on their study of a surface treatment of hematite photoanodes using zinc acetate for water oxidation [37]. The results further reveal that the surface doping with Ag and Zn did not change the hematite from the photoanode to photocathode hence, the oxygen evolution reaction (OER) still took place on the photoelectrodes.

**Figure 10:**
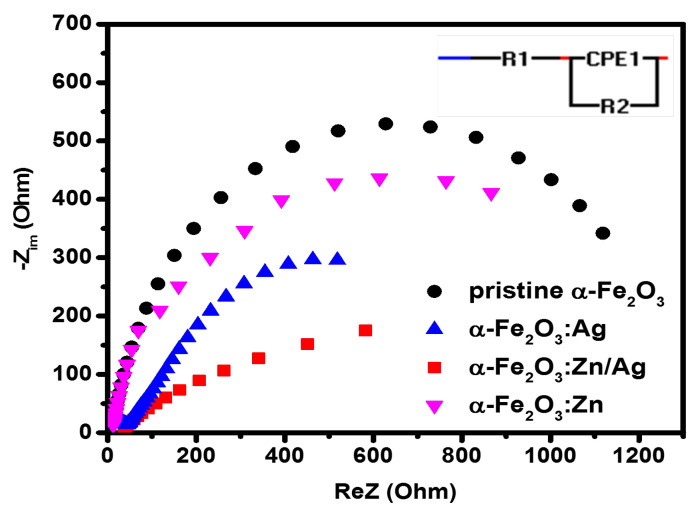
Electrochemical impedance measurements of pristine α-Fe${}_{2}$O${}_{3}$, α-Fe${}_{2}$O${}_{3}$:Zn, α-Fe${}_{2}$O${}_{3}$:Ag and α-Fe${}_{2}$O${}_{3}$:Zn/Ag using a 1 M NaOH electrolyte under illumination, with the inset showing an equivalent circuit. R1 and R2 are the resistance of FTO and charge transfer, respectively, while CPE1 is the constant phase element that defines the charge gradient.

To better understand the electrochemical behavior of these photoanodes, a three-electrode configuration was used to measure the electrochemical impedance. The impedance measurements were determined using Ohms law,

(5) $$Z\left(\omega \right)=\frac{V(t)}{I(t)},$$

where *V(t)* = *V${}_{o}$* + *V${}_{m}$*sin(*ωt*), and *I(t)* = *I${}_{o}$* + *I${}_{m}$*sin(*ωt* + θ). Here, *V${}_{o}$* and *I${}_{o}$* represent the dc bias potential and a steady-state current flowing in the electrolyte when performing the EIS experiment, while the maximum obtained voltage and sinusoidal current signal are represented by *V*${}_{m}$ and *I*${}_{m}$, respectively, while θ is the phase angle [38]. The EIS Nyquist plots of the pristine hematite, α-Fe${}_{2}$O${}_{3}$:Zn, α-Fe${}_{2}$O${}_{3}$:Ag, and α-Fe${}_{2}$O${}_{3}$:Zn/Ag samples are presented in Figure 10. The curve for pristine hematite exhibits the largest diameter while that of α-Fe${}_{2}$O${}_{3}$:Zn/Ag showed the smallest diameter. Besides, the n-type α-Fe${}_{2}$O${}_{3}$/electrolyte for a PEC system is normally presented by the largest semicircle [39], which was in agreement with our results. This also indicates that there was high resistance in the undoped hematite compared to the doped ones (α-Fe${}_{2}$O${}_{3}$:Zn, α-Fe${}_{2}$O${}_{3}$:Ag, and α-Fe${}_{2}$O${}_{3}$:Zn/Ag). In addition, this smaller semicircle further explains the improvement in the photocurrent for α-Fe${}_{2}$O${}_{3}$:Zn/Ag samples, as compared to pristine α-Fe${}_{2}$O${}_{3}$ [25]. Doping, therefore, reduces the OER barrier by reducing the charge transfer barrier at the electrode interface [40].

**Figure 11:**
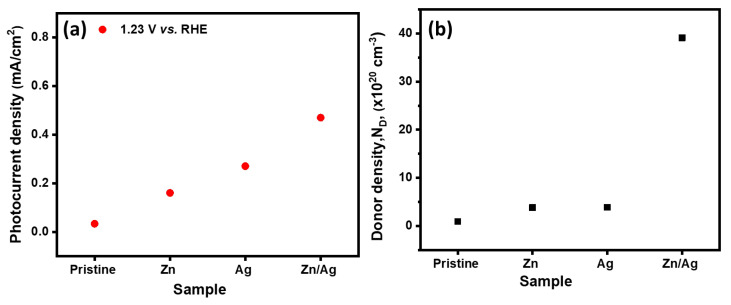
(**a**) Comparison of the photocurrent density, at 1.23 V vs. RHE, and (**b**) donor density (*N*${}_{D}$) of pristine α-Fe${}_{2}$O${}_{3}$, α-Fe${}_{2}$O${}_{3}$:Zn, α-Fe${}_{2}$O${}_{3}$:Ag, and α-Fe${}_{2}$O${}_{3}$:Zn/Ag.

The equivalent electrical circuit models that give more information on the charge trapping, transport, and charge transfer at the electrolyte/photoelectrode interface were performed using EIS spectrum analyzer software [41]. The RC circuit consisting of FTO resistance (R1), a constant phase element (CPE1), and the charge transfer resistance, R2, (R${}_{ct}$) at the α-Fe${}_{2}$O${}_{3}$/electrolyte was successfully used to fit the data for all our samples. From the fitting results, there was a reduction in the charge transfer resistance of all the doped samples, compared to pristine hematite. The high resistance on the semiconductors from this study can be assigned to the α-Fe${}_{2}$O${}_{3}$/electrolyte interface. This is due to the difficulties the minority charge carriers (holes) experience to travel from the hematite layer to oxidize water, mainly due to the poor conductivity of hematite [39]. The lowest R${}_{ct}$ was observed on the α-Fe${}_{2}$O${}_{3}$:Zn/Ag sample, which agrees with the photocurrent results. At the same time, there was an observable change in CPE1 for the doped samples compared to that of pristine α-Fe${}_{2}$O${}_{3}$, which further explains the small semicircle obtained for those photoelectrodes. Additionally, the decrease in CPE1 indicates the formation of a high charge transfer gradient when the dopants are introduced. Figure 11 summarizes the comparison of the photocurrent density at 1.23 V vs. RHE, and the donor densities while (Table 1) presents the resistance and capacitance fitting values for Nyquist plots for undoped and doped hematite.

### 3.6. Ultrafast Transient Absorption Spectroscopy

**Figure 12:**
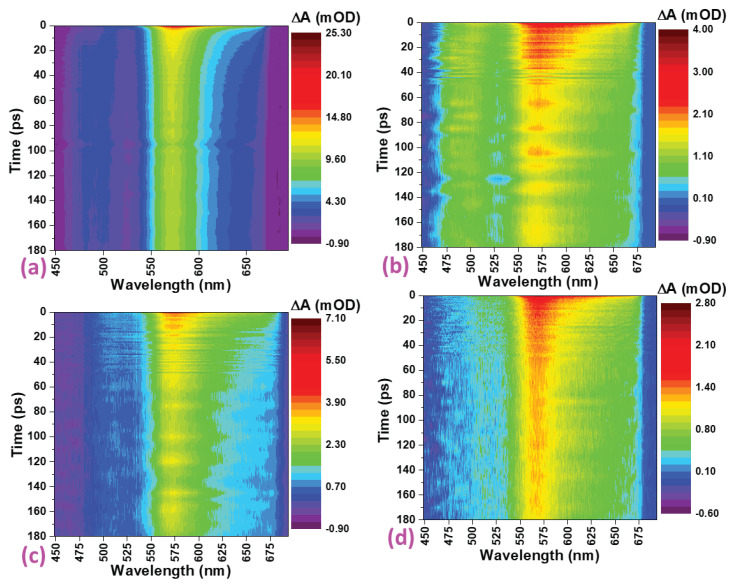
Difference absorption (△A) as a function of delay time and wavelength for (**a**) Pristine α-Fe${}_{2}$O${}_{3}$ (**b**) α-Fe${}_{2}$O${}_{3}$:Zn, (**c**) α-Fe${}_{2}$O${}_{3}$:Ag and (**d**) α-Fe${}_{2}$O${}_{3}$:Zn/Ag.

**Figure 13:**
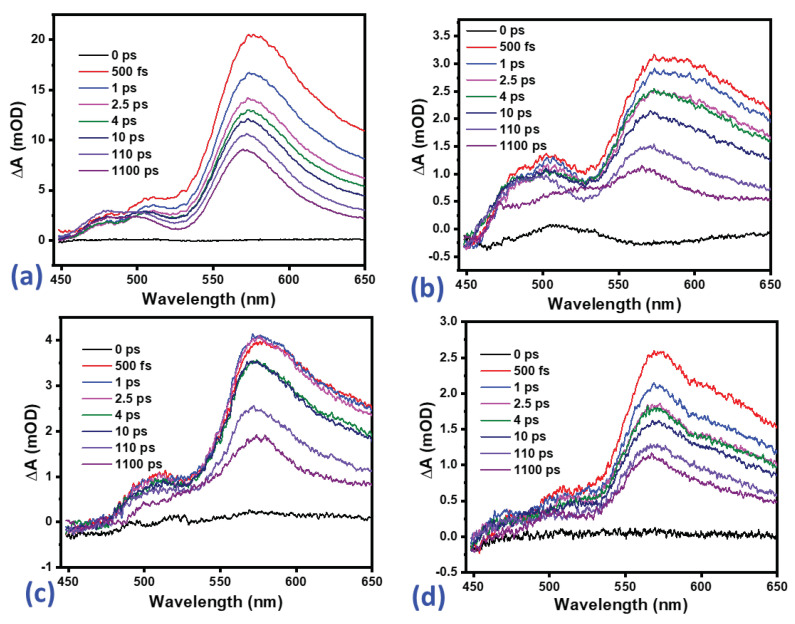
Difference absorption spectra at selected delay times for (**a**) pristine α-Fe${}_{2}$O${}_{3}$ (**b**) α-Fe${}_{2}$O${}_{3}$:Zn, (**c**) α-Fe${}_{2}$O${}_{3}$:Ag and (**d**) α-Fe${}_{2}$O${}_{3}$:Zn/Ag samples measured using transient ultrafast spectroscopy.

Colour maps of the time and wavelength-dependence of the difference absorption (△A) of pristine α-Fe${}_{2}$O${}_{3}$, α-Fe${}_{2}$O${}_{3}$:Zn, α-Fe${}_{2}$O${}_{3}$:Ag and α-Fe${}_{2}$O${}_{3}$:Zn/Ag obtained from ultrafast transient absorption spectroscopy are presented in Figure 12 for delay times up to 180 ps and line spectra at selected delay times are shown in Figure 13. The broad, positive bands in Figure 12 and Figure 13 are centered near 570 nm, extend beyond 650 nm and relate to excited state absorption (ESA). The heat maps (Figure 12) describe the changes in the ESA feature. For all samples, this ESA band was present from zero delay time, indicating rapid formation within the ultrashort time resolution of our setup, and decays slowly, showing a significant amplitude still after 1.1 ns. Although the doping does not necessarily improve the lifetimes of the charge carriers as reported previously by Morris and co-workers [42], the morphology of the samples synthesized had a great impact since the average grain size of α-Fe${}_{2}$O${}_{3}$:Zn, α-Fe${}_{2}$O${}_{3}$:Ag and α-Fe${}_{2}$O${}_{3}$:Zn/Ag samples was <30 nm.

**Figure 14:**
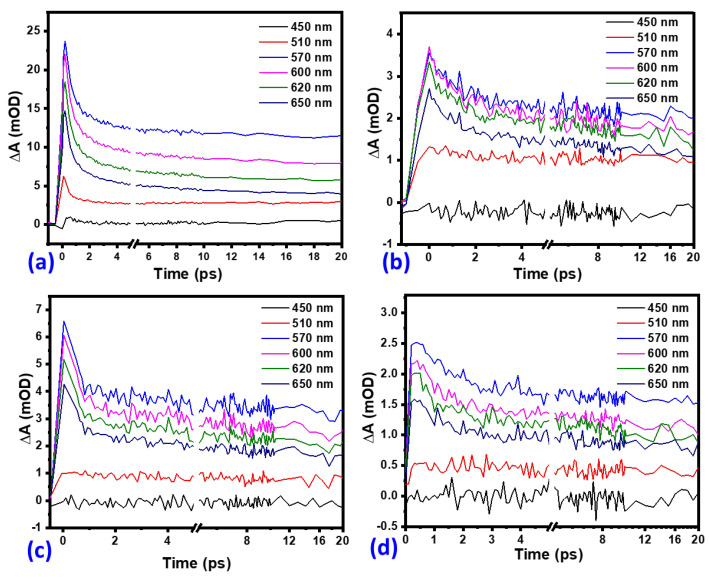
Kinetic traces at selected wavelengths (450, 510,570, 600, 620, and 650 nm) for (**a**) pristine α-Fe${}_{2}$O${}_{3}$, (**b**) α-Fe${}_{2}$O${}_{3}$:Zn, (**c**) α-Fe${}_{2}$O${}_{3}$:Ag, and, (**d**) α-Fe${}_{2}$O${}_{3}$:Zn/Ag.

**Figure 15:**
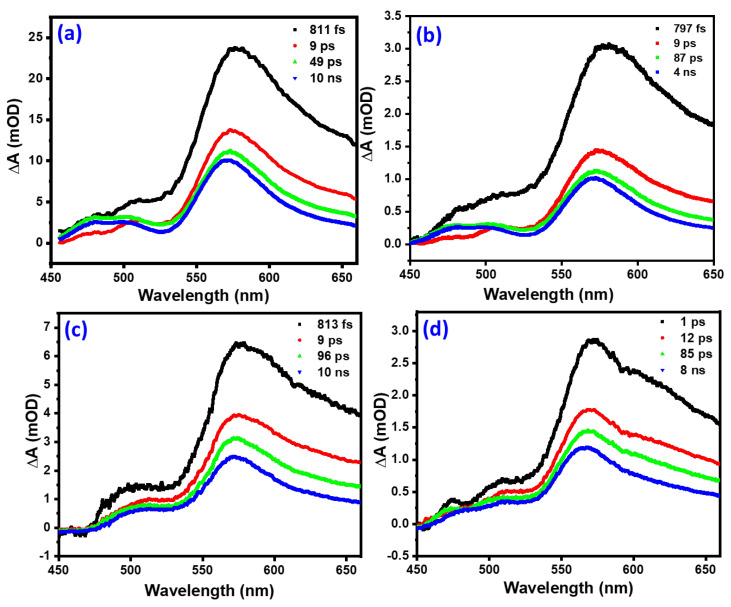
Evolution associated difference spectra (EADS) with associated decay lifetimes for (**a**) pristine α-Fe${}_{2}$O${}_{3}$, (**b**) α-Fe${}_{2}$O${}_{3}$:Zn, (**c**) α-Fe${}_{2}$O${}_{3}$:Ag, and, (**d**) α-Fe${}_{2}$O${}_{3}$:Zn/Ag.

To further explain the electron-hole recombination resulting from the decay of the positive band (570 nm peak), kinetic traces at selected wavelengths in the range of 450 to 650 nm were extracted as shown in Figure 14. There was no observable ground-state bleach of the four samples as shown in Figure 14 and Figure 15. The presence of intra-bandgap states in hematite thin films has also been associated with the ESA feature [43]. On the other hand, a study conducted by Sorenson and co-workers on the comparison of transient absorption signals by calculation of the hematite electronic band structure concluded that the positive band could result from excited-electron absorption [44]. In Figure 13, the signals can be observed to have grown to the maximum value in the transient time scale (<150 fs) and decayed faster for the first 2 ps before slowing down.

### 3.7. Global Analysis

Global analysis was performed to determine the electron-hole dynamics (e-h relaxation to the respective band edges, electron trapping by defects and e-h recombination) for pristine α-Fe${}_{2}$O${}_{3}$, α-Fe${}_{2}$O${}_{3}$:Zn, α-Fe${}_{2}$O${}_{3}$:Ag, and α-Fe${}_{2}$O${}_{3}$:Zn/Ag. This was done using a four-component sequential model described elsewhere [16]. The evolution-associated difference spectra (EADS) with associated decay lifetimes for the pristine α-Fe${}_{2}$O${}_{3}$, α-Fe${}_{2}$O${}_{3}$:Zn, α-Fe${}_{2}$O${}_{3}$:Ag, and α-Fe${}_{2}$O${}_{3}$:Zn/Ag samples were obtained from the analysis and are shown in Figure 15. These lifetimes ranged from femtosecond to nanosecond timescales.

During the process of excitation from the ground to excited states, creation of hot electrons and holes takes place. The electrons later thermalize and relax to the bottom of the conduction band while the holes relax to the top of the valence band, and most of the process happens on a sub-ps timescale [45]. This process was associated with the first lifetime, τ${}_{1}$ (Table 2) ranging between 797 fs and 1 ps for the four samples. The variation of the lifetimes could be due to the improvement in the conductivity that resulted from the monodoping and codoping. This is illustrated in Figure 9 where there was an increase in the donor density of the codoped and monodoped from the pristine hematite. This means that there was a faster charge transport on the doped samples than pristine hematite. The τ${}_{2}$ lifetimes obtained in this study ranged from 9 to 12 ps. Hematite is known to have a high density of midgap states, the occupancy of which takes place on a femtosecond-picosecond timescale. Therefore, τ${}_{2}$ is associated with electron trapping by midgap states. The τ${}_{3}$ lifetimes that ranged from 49 to 97 ps can be assigned to the direct recombination of electrons and holes trapped by the midgap states, leading to a longer lifetime than the pure trap-assisted recombination [16]. The doped samples have basically the same lifetime that is significantly longer (almost double) than that of pristine hematite. However, the prolongation in lifetime observed in τ${}_{3}$ compared to pristine hematite could be due to the nanorod-like structures that were obtained as shown in the FESEM micrographs (Figure 5). The τ${}_{2}$ and τ${}_{3}$ lifetimes obtained in this study support the Mott–Schottky measurements (Figure 9). The signals related to low trap state densities could have been present on the Mott–Schottky plots even though hematite suffers from poor conductivity that results from the valence alternation of Fe${}^{3+}$/Fe${}^{2+}$ on spatially localized 3d orbitals, affecting the efficiency of charge separation [46]. This could result from the strong coupling between the conduction band and the trap states. Additionally, oxygen vacancies, which are the primary defects in hematite, might have led to a high density of these trap states [47]. There was not much change observed in the XRD structure of hematite during doping. However, the variation in these lifetimes for α-Fe${}_{2}$O${}_{3}$:Zn, α-Fe${}_{2}$O${}_{3}$:Ag, and α-Fe${}_{2}$O${}_{3}$:Zn/Ag compared to that of pristine α-Fe${}_{2}$O${}_{3}$ could be due to differences in the grain sizes. The fourth lifetime was on a ns timescale. One possible cause of these long lifetimes is the recombination between valence band holes and those electrons previously transferred to the FTO and transferred back to hematite through backflow [16]. This is possible due to the 0.19 eV energetic difference between FTO and hematite [11,16]. More studies on the other possible causes of the fourth lifetime still need to be done. From Figure 15a, there seems to be an interplay between 505 nm and 570 nm bands, while Figure 15b–d show a blue-shift of the main band with time. From this study, it can be concluded that the shape of the nanostructures does not affect the lifetime of the samples on the picosecond-nanosecond timescale which is similar to what has been reported by other researchers [42,48]. Table 2 gives a summary of the current density (J), donor density, and lifetimes obtained from this study.

## 4. Conclusions

Pristine α-Fe${}_{2}$O${}_{3}$, α-Fe${}_{2}$O${}_{3}$:Zn, α-Fe${}_{2}$O${}_{3}$:Ag, and α-Fe${}_{2}$O${}_{3}$:Zn/Ag were successfully synthesized using spray pyrolysis. X-ray diffraction studies confirmed hematite with the corundum structure with an R$\bar{3}$C space group with *a* = *b* = 5.075 Å and *c* = 13.748 Å lattice parameters. The main peaks (110) and (104) indexed at $33.3^{{}^{\circ}}$ and $35.5^{{}^{\circ}}$ were observed in all samples. There was a slight shift of the main phases of hematite for doped samples due to an increasing ionic radius of Fe${}^{3+}$ by Ag${}^{+}$ and Zn${}^{2+}$. Bandgaps of pristine hematite, α-Fe${}_{2}$O${}_{3}$:Zn, α-Fe${}_{2}$O${}_{3}$:Ag and α-Fe${}_{2}$O${}_{3}$:Zn/Ag were obtained as 1.9, 2.1, 2.0 and 2.1 eV, respectively. Mott–Schottky confirmed the n-type conductivity of all the samples. An increase in donor density of more than one order of magnitude from 1.57 × 10${}^{19}$ to 8.75 × 10${}^{20}$ cm${}^{-3}$ for pristine α-Fe${}_{2}$O${}_{3}$ and α-Fe${}_{2}$O${}_{3}$:Zn/Ag, respectively, was observed. This led to an improvement in the current density for the doped samples compared to pristine α-Fe${}_{2}$O${}_{3}$. α-Fe${}_{2}$O${}_{3}$:Zn/Ag revealed the lowest impedance while pristine hematite had the largest value, a further indication of low resistance for the surface doped samples as compared to the undoped one. We conclude from this that doped samples had an enhanced photocurrent, with α-Fe${}_{2}$O${}_{3}$:Zn/Ag producing the highest photocurrent of 0.470 from 0.033 mAcm${}^{-2}$ at 1.23 V vs. RHE for pristine α-Fe${}_{2}$O${}_{3}$.

Transient absorption spectroscopy was performed to obtain transient spectral signatures and four associated decay lifetimes of all the samples. The first sub-picosecond lifetime was associated with thermalization of excited electrons. The τ${}_{2}$ lifetimes obtained in this study is assigned to electron trapping by midgap states. The τ${}_{3}$ lifetimes, on the other hand, is assigned to direct electron-hole recombination trapped by the midgap states, leading to a longer lifetime than the pure trap assisted recombination. Finally, the fourth, nanosecond lifetime was proposed to result from recombination of electrons and holes transferred from the back of the FTO substrate. This study provides a deeper understanding of the ultrafast spectroscopic studies of electron-hole recombination rates of mono-doping and co-doping of hematite for improvement in PEC performance. The changes in the resolved lifetimes obtained from this study were consistent with the improvement in the photoactivity for the doped samples. Since there was a significant improvement in hydrogen production, future work will explore L-arginine/surface mono-doped and co-doped hematite thin films, with the aim of further photoactivity enhancement.

## Figures and Tables

**Table 1:** Resistance and capacitance fitting values for Nyquist plots of the samples.

Sample	R1	CPE1	R2
	(Ω)	(×10${}^{-6}$ Fcm${}^{-2}$)	(Ω)
pristine hematite	57.9	7.7	1162.3
α-Fe${}_{2}$O${}_{3}$:Zn	53.2	7.5	808.5
α-Fe${}_{2}$O${}_{3}$:Ag	50.4	7.4	753.5
α-Fe${}_{2}$O${}_{3}$:Zn/Ag	50.3	4.2	749.8

**Table 2:** Summary of current density and lifetimes of pristine and doped samples obtained from global analysis.

Sample	J (1.23V)	τ ${}_{1}$	τ ${}_{2}$	τ ${}_{3}$	τ ${}_{4}$	Donor Density
	(mAcm${}^{-2}$)	(fs)	(ps)	(ps)	(ns)	(×10${}^{20}$ cm${}^{-3}$)
Pristine hematite	0.033	811	9	49	10	0.157
α-Fe${}_{2}$O${}_{3}$:Zn	0.160	797	9	97	4	5.63
α-Fe${}_{2}$O${}_{3}$:Ag	0.270	813	9	96	10	6.91
α-Fe${}_{2}$O${}_{3}$:Zn/Ag	0.470	1005	12	85	8	8.75

## Data Availability

The data used and or analysed during the current study are available from the corresponding author upon request.
